# Helicase HELQ: Molecular Characters Fit for DSB Repair Function

**DOI:** 10.3390/ijms25168634

**Published:** 2024-08-08

**Authors:** Yuqin Zhao, Kaiping Hou, Yu Liu, Yinan Na, Chao Li, Haoyuan Luo, Hailong Wang

**Affiliations:** Beijing Key Laboratory of DNA Damage Response, College of Life Sciences, Capital Normal University, Beijing 100048, China

**Keywords:** HELQ, biochemical function, DSB repair, end resection

## Abstract

The protein sequence and spatial structure of DNA helicase HELQ are highly conserved, spanning from archaea to humans. Aside from its helicase activity, which is based on DNA binding and translocation, it has also been recently reconfirmed that human HELQ possesses DNA–strand–annealing activity, similar to that of the archaeal HELQ homolog StoHjm. These biochemical functions play an important role in regulating various double–strand break (DSB) repair pathways, as well as multiple steps in different DSB repair processes. HELQ primarily facilitates repair in end–resection–dependent DSB repair pathways, such as homologous recombination (HR), single–strand annealing (SSA), microhomology–mediated end joining (MMEJ), as well as the sub-pathways’ synthesis–dependent strand annealing (SDSA) and break–induced replication (BIR) within HR. The biochemical functions of HELQ are significant in end resection and its downstream pathways, such as strand invasion, DNA synthesis, and gene conversion. Different biochemical activities are required to support DSB repair at various stages. This review focuses on the functional studies of the biochemical roles of HELQ during different stages of diverse DSB repair pathways.

## 1. Introduction

DNA is the fundamental template for replication and transcription, serving as the cornerstone for maintaining genome stability and the continuation of life. However, DNA is susceptible to various types of damage, among which DNA double–strand breaks (DSBs) are particularly severe due to their potential to cause chromosomal fragmentation and genetic instability. Organisms have evolved sophisticated mechanisms to repair DSBs, ensuring genomic integrity. Among these repair mechanisms, the role of helicases, particularly HELQ helicase, has garnered significant attention.

HELQ proteins are part of the Mus308 subfamily, which belongs to the ske2–like family within the SF2 family. This family is the largest helicase superfamily found in almost all living organisms. From archaea to humans, these proteins are named differently in different species. For example, Hel308 (*Archaeoglobus fulgidus* and *Sulfolobus solfataricus*) and Hjm (*Pyrococcus furiosus*) are identified in archaea, Helq-1 in eukaryotic *Caenorhabditis elegans*, Mus301 in *Drosophila melanogaster*, and now HELQ, HelQ, Hel308, or Helq in *Homo sapiens* [1].

The discovery of HELQ dates back to 1990, when Paul V. Harris and his team identified the Mus308 gene in *Drosophila melanogaster*, noting its sensitivity to DNA cross–linking agents, such as nitrogen mustard, trimethylpsoralen, dioxobutane, and cisplatin [2]. Subsequent research by Kenneth C. Burtis et al. in 1996 found a similar gene in *Caenorhabditis elegans*, encoding a polypeptide with both DNA polymerase and helicase activities [3]. In 2002, Richard D. Wood et al. identified the mammalian homolog HEL308, which is capable of translocating along DNA in a 3′–5′ direction and behaving as a multimer [4]. Further studies by Yoshizumi Ishino et al. demonstrated that the Hjm protein in archaea shares significant sequence similarity with human HEL308, Drosophila Mus301, and human PolQ and possesses DNA helicase activity. However, they did not find similar sequences in bacteria and yeast [5].

The helicase activity of HELQ is crucial for unwinding the lagging strand of the replication fork, which facilitates the restart of lagging strand synthesis. Besides its helicase activity, HELQ exhibits DNA–binding capabilities essential for DNA unwinding, DSB repair, and the protection of stalled replication forks from degradation [6,7,8,9]. Additionally, studies by Anand et al. have demonstrated HELQ’s ability to anneal DNA strands [8], which aids in DSB repair and prevents gene conversion [8,10].

Despite extensive research on HELQ, a comprehensive review that systematically summarizes how its biochemical activities are adapted for DSB repair is lacking. This review aims to fill that gap by providing a systematic survey of recent studies on HELQ, emphasizing the biochemical insights into its role in DSB repair. This study aims to elucidate the significance of HELQ in maintaining genomic stability and highlight its potential as a target for therapeutic interventions in diseases associated with DNA damage.

## 2. Molecular Architecture of HELQ Family Proteins

### 2.1. The HELQ Protein Sequence Is Conserved from Archaea to Humans

**Figure 1:**
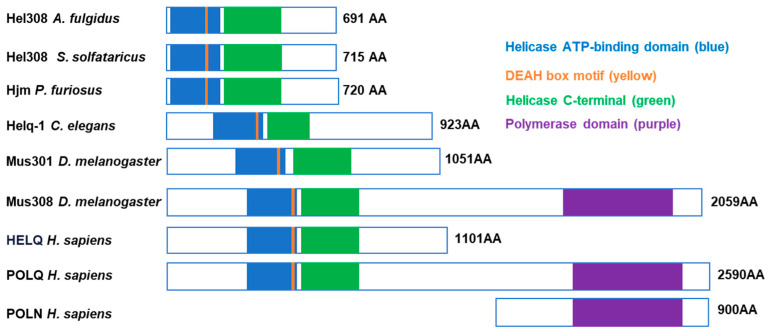
Schematic diagram of selected members of MUS308 subfamily 2 of DNA helicases. The helicase domain includes the helicase ATP–binding domain (blue) and helicase C terminal (green). The polymerase domain of MUS308 and POLQ are depicted in purple, and the DEAH box motif is shown in yellow.

At first glance, the differences in the primary sequences of HELQ orthologs are remarkable. However, it has been found that HELQ orthologs share about 30% sequence identity after bioinformatic alignment between *Homo sapiens* and *Archaeoglobus fulgidus*. This indicates a relatively high degree of protein sequence similarity. Notably, these proteins exhibit a highly conserved helicase domain from archaea to eukaryotes [1,11]. All of the proteins have the Helicase ATP–binding domain, DEAH box motif, and Helicase C–terminal domain (Figure 1). Human *HELQ* has 40% identity and 55% similarity in the helicase domain with the *Drosophila Mus308* and *Mus308* human homologous gene *POLQ* [1,11]. POLQ contains 2590 amino acids and belongs to the DNA polymerase family, which includes both a helicase domain and a polymerase domain. However, human HELQ contains only a helicase domain. Interestingly, POLQ’s polymerase domain is highly conserved with the human DNA polymerase POLN [1,11,12] (Figure 1). The conserved nature of HELQ homologs across different species also maintains their unique biological functions throughout evolution.

### 2.2. The HELQ 3–D Spatial Structure Is Conserved from Archaea to Humans

HELQ orthologs from different species not only exhibit high sequence homology but also share a high degree of similarity in protein spatial structure. Buttner et al. were the first to resolve the crystal structure of Hel308 in *Archaeoglobus fulgidus* archaea in 2007, achieving a resolution of 3.1 Å (Angstroms) [13]. To explore the mechanism by which DNA captures Hel308 during initial strand separation, they also resolved the crystal structure of a 3′ overhang DNA substrate bound to Hel308. Their findings demonstrate that ATP is not required for the initiation of strand separation and that the unwinding direction is 3′–5′. The crystal structure reveals that Hel308 consists of five domains (Domains 1–5) and a β–hairpin ring. Domains 1 and 2 (RecA–fold domain) contain ATP–binding sites, while Domain 3 (Winged–helix domain, WHD) features a winged–shaped spiral folding, tightly packed with Domain 1. Domain 4 (Ratchet domain) consists of a seven–helix bundle that, together with Domains 1 and 3, forms a loop around the 3′ DNA tail. Domain 5 (HLH domain) is a helix–loop–helix structure with a loop located at the periphery of the ring, binding the distal end of the 3′ DNA tail. The prominent β–hairpin ring, including amino acids R350, F351, Y354, and R357, acts as an unwinding element, stretching out the DNA at the opening. Similar β–hairpin loops are found in hepatitis C virus NS3 helicase and RNA degradation factors. This double–stranded unwinding mechanism is widely applicable to SF2–family helicases. Except for some slight shifts in Domain 5 and minor changes in DNA and ATP–binding groups in the presence of DNA, the overall structure of Hel308 remains consistent with and without DNA [13].

Richards et al. aligned the crystal structure of *Sulfolobus solfataricus* Hel308 with that of *Archaeoglobus fulgidus* Hel308 and found no significant changes in structure, indicating that this helicase maintains a rigid structure for its unwinding function [7]; Oyama et al. also supported this conclusion [14].

**Figure 2:**
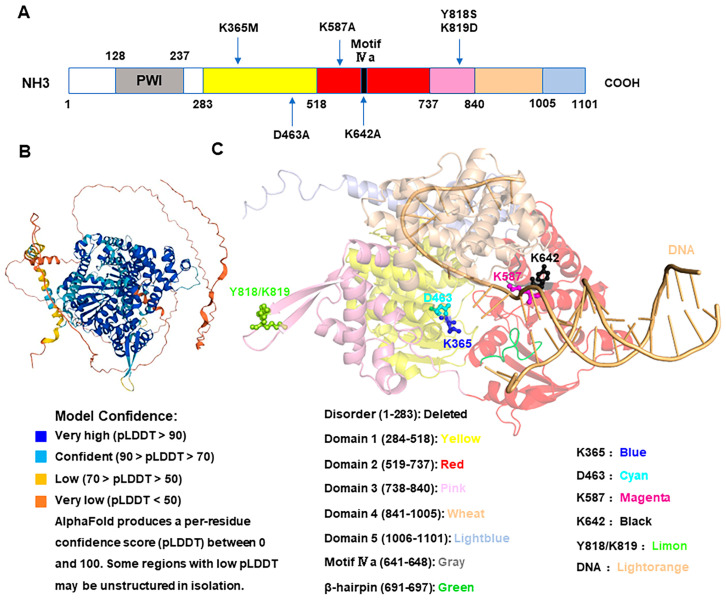
Structural diagram of HELQ protein. (**A**) Protein (amino acid) information of human *HELQ* gene. (**B**) The human HELQ protein structure is predicted by AlphaFold. Overall, the pLDDT values for amino acids 1–314 are less than 70, while those for 315–1101 are greater than 70. K365, D463, K587, and K642 are all located within the region where the pLDDT is greater than 90, and the pLDDT of Y818/K819 is between 50 and 90. (**C**) Structure and domain organization of human HELQ acquired by alignment with Hel308 from *Archaeoglobus fulgidus* and *Sulfolobus solfataricus*. Illustration showing the five domains and a β–hairpin arrangement of HELQ binding to DNA [13]. The DNA is from the crystal structures *Archaeoglobus fulgidus* Hel308 in complex with DNA. Sticks indicate the functional amino acid sites. K365, ATP–binding site; D463, ATP–hydrolysis site; K587, ssDNA–binding site; K642, translocating site; Y818/K819, dsDNA–binding sites.

Although the crystal structure of human HELQ has not been resolved, a bioinformatic analysis indicates high conservation among HELQ homologs across different species. Using AlphaFold and PYMOL, the spatial structure of human HELQ can be predicted and aligned with the resolved structures of *Archaeoglobus fulgidus* and *Sulfolobus solfataricus*. The predicted human HELQ structure was found to be highly similar to *Archaeoglobus fulgidus* Hel308, with an RMSD value of 3.083 Å (*Angstroms*) [9], including five domains and a β hairpin (Figure 2).

In summary, the structure of the HELQ protein is highly conserved from archaea to humans, facilitating the extension of its biological function from lower to higher organisms.

## 3. The Biochemical Function of HELQ

### 3.1. DNA Unwinding

The structure of HELQ orthologs reveals not only a high degree of sequence homology but also a significant similarity in protein spatial structure. Buttner et al. (2007) first resolved the crystal structure of Hel308 in Archaeoglobus fulgidus archaea, achieving a resolution of 3.1 Å. Specifically, the Hel308–DNA co-structure reveals extensive molecular interactions between Hel308 and ssDNA. This groundbreaking work provided detailed insights into the mechanism of DNA unwinding, revealing that ATP is not required for the initiation of strand separation and that the unwinding direction is 3′–5′ [13].

The Hel308 helicase is powered by ATP (binding to Domain 2), aided by the *WHD* (Domain 3) binding to dsDNA. The “ratchet” spiral in Domain 4 is utilized to gradually translocate the ssDNA from 3′ to 5′. The β–hairpin loop serves to open the newly unwound DNA to prevent it from re–annealing, while the braking mechanism in Domain 5 effectively regulates the entire unwinding process. The conformational movements guided by the unwinding mechanism are linked with physical phenomena [13,15,16]. The atomic resolution structure of Hel308 provides valuable assistance for further study of its biochemical functions.

ATP energy is utilized by helicases as a motor to unwind DNA. The archaeal *Sulfolobus solfataricus* Hel308 K52 (now K59) residue exhibits ATPase and helicase activities [7]. The K365 residue of Homo sapiens HELQ is crucial for ATP binding [12], and the K463 residue is essential for ATP hydrolysis [17] (Figure 2). Disruption of these critical residues results in the loss of DNA–unwinding capability.

### 3.2. DNA Binding

DNA binding is fundamental to the helicase activity of HELQ, as it ensures the helicase remains attached to the DNA substrate during the unwinding process, preventing detachment and delays. HELQ has been demonstrated to be an ssDNA–activated ATPase, essential for unwinding various DNA structures such as forked DNA, ssDNA, and dsDNA junctions, as well as 3′ overhangs, 3′ lagging strand forks, Y–structures, and D–loops [4,18]. However, HELQ cannot unwind ATPγS or 5′ overhang substrates [4,8,18]. It has been reported that HELQ has a stronger preference for ssDNA binding than dsDNA [7,8]. 

Richards et al. identified three conserved arginine residues (R255, R320, and R662) in *Sulfolobus solfataricus* Hel308 that bind to DNA, with R255 showing the strongest binding to ssDNA [7]. This residue is also conserved in *Archaeoglobus fulgidus* Hel308 (R252) [13] and Homo sapiens HELQ K587 (Figure 2) [9], and mutations at this site can significantly impair its unwinding ability. Conversely, the helicase activity mutant K52A (now K59) impairs unwinding but does not affect ssDNA and dsDNA affinity [7,8], which suggests that the ability to bind to DNA is critical for the HELQ’ unwinding process.

Furthermore, the WHD binds to dsDNA but not to ssDNA. The dsDNA binding sites in Homo sapiens HELQ are Y818–K819 (WHD Y–K) (Figure 2) [15], and mutations in the WHD reduce dsDNA binding and unwinding but do not affect ssDNA binding [15], suggesting that the ability to bind to dsDNA is also vital for its unwinding.

The helicase activity of HELQ is also regulated by other proteins. For example, the HELQ unwinding 3′ overhang substrates are inhibited by RPA, whereas RAD51 stimulates the unwinding activity of its D–loops by forming a complex with HELQ [8]. On the contrary, Jenkins et al. proposed that HelQ mediates interactions with RPA, which coordinates the loading of the helicase domains onto ssDNA by the PWI–like domain (amino acids 128–237 of the non-catalytic N–terminal region of HelQ). Once HelQ is loaded onto the ssDNA, ATP–Mg^2+^ binding in the catalytic site activates the helicase core, disrupts RPA–ssDNA complexes through conserved Asp–141 and Phe–142 residues, and triggers translocation along ssDNA and unwinding as a dimer [17]. The reason for the discrepancy in the results of the two reports requires further explanation. This discrepancy may be due to the fact that RPA competes with HELQ during the regulation of DNA unwinding, or different regions of HELQ interact with RPA, playing various roles in regulating the unwinding function of HELQ, which leads to this discrepancy.

Overall, the unwinding activity is influenced by various factors, including but not limited to ssDNA– and dsDNA–binding abilities or some other proteins.

### 3.3. DNA Translocating

Besides its capacity to bind DNA, HELQ also requires translocation activity to effectively unwind DNA. The *Archaeoglobus fulgidus* Hel308 atomic–resolution structure provides detailed information on the mechanism of ssDNA translocation in unwinding DNA [4]. The α–helical “ratchet” Domain 4 has been identified as essential for DNA translocation [10]. In addition, Jenkins et al. theorized that the Homo sapiens HelQ Y642 residue (Figure 2) is conserved in a motif of Domain 2 in Ski2 helicases, which is required for DNA translocation. A mutation of HelQ Y642 to Y642A impairs the unwinding capability, underscoring its essential role in DNA translocation [17].

This translocation capability is crucial for removing proteins bound to DNA. For example, Richards et al. demonstrated that Hel308 could displace streptavidin from biotinylated DNA substrates [7]. Similarly, Jenkins and Anand et al. found that Hel308 could remove RPA from ssDNA [8,17]. The translocation activity of HELQ is also harnessed in the Nanopore sequencing of DNA and peptides [19,20].

The above–described findings suggest that one function of HELQ is to remove bound proteins from stalled replication forks and recombinant intermediates, thereby maintaining genomic stability. DNA–binding activity is essential for translocation along ssDNA, and the ability to translocate is necessary for unwinding. Therefore, the helicase activity of HELQ depends on its DNA–binding and translocation abilities.

### 3.4. DNA Annealing

The ability of HELQ to facilitate DNA annealing was first observed in the archaea *Sulfolobus tokodaii*, where a related protein, StoHjm, was shown to unwind DNA in both the 3′–5′ and 5′–3′ directions. In vitro, studies revealed that StoHjm can unwind both the leading and lagging strands of the replication fork, demonstrating its capacity for structure–specific activity that promotes single–strand annealing and contributes to DNA replication fork reversal [21]. In contrast, the HELQ analog generally exhibits ATP–dependent 3′–5′ unwinding activity. However, recent findings indicate that Homo sapiens and *Methanothermobacter thermautotrophicus* HELQ also have DNA–annealing abilities [8,10]. 

A highly conserved motif (motif IVa, AF/YHHAGL) [4,18] of the RecA2 domain of the Hel308/HELQ helicase has been reported to possess annealing abilities. This motif is conserved in the superfamily 2 helicase of the Hel308, Ski2, and RecQ subfamilies, but its function is unknown [6]. In 2023, Lever et al. discovered that IVa can regulate both the DNA–unwinding and –annealing functions in *Methanothermobacter thermautotrophicus* Hel308. Purified Hel308 from this organism, with a single amino acid substitution (F295A) in motif IVa, exhibited hyperactive DNA–binding, –unwinding, and –annealing activities. This activity is also ATP–dependent [10].

Interestingly, the *Methanothermobacter thermautotrophicus* Hel308 F295 residue and the Homo sapiens HelQ/HELQ Y642 residue are conserved based on sequencing alignment and are located in motif IVa, suggesting a functional relationship. The Homo sapiens HELQ motif IVa consists of amino acids 641–648, according to sequencing alignment (Figure 2); therefore, the Homo sapiens HelQ/HELQ Y642A mutant should promote unwinding. It is worth considering why these residues have completely opposite effects for unwinding. Additionally, the β–hairpin loop could open the newly unwound DNA to prevent the unwinding DNA from re–annealing; therefore, a mutated β–hairpin loop may promote annealing.

Furthermore, other factors influence HELQ’s DNA–annealing abilities. Tafel et al. noted that Homo sapiens HEL308/HELQ does not exhibit a strong annealing activity and that RPA directly stimulates the helicase activity of HEL308 rather than inhibiting strand re–annealing [18]. Anand et al. detected that HELQ showed no DNA annealing without ATP in the presence of RPA. HELQ’s ATP–binding site mutant K365M also cannot complete DNA annealing, whereas ATP becomes dispensable when RPA is excluded from the reaction [8]. Overall, it has been demonstrated that HELQ has an intrinsic ability to capture the DNA strand bound to RPA and then displaces the RPA to facilitate annealing in vitro [8], which suggests that HELQ can promote DNA annealing under certain conditions. Liu He et al. further proposed that HelQ/HELQ strongly stimulates HelQ–mediated DNA single–stranded annealing through physical interactions with POLD3 via 70 amino acids (amino acids 1–76) in its N–terminal disordered region [22].

In conclusion, different regions of HELQ interact with distinct proteins, leading to various regulatory effects on its annealing activity. Therefore, the roles of HELQ’s ssDNA–binding site at K587, the translocating site at Y642, and the ATP hydrolysis site at K463 in annealing should be investigated.

## 4. Biochemical Activity Fit for DSB Repair Function

### 4.1. DSB Repair

DNA is a fundamental template for replication and transcription, serving as the bedrock for genome stability and the continuity of life. The role of DNA is crucial, yet it is vulnerable to various forms of damage during continuous use and transmission. While some types of damage, such as oxidative modifications to DNA bases [23], are relatively common, DSBs are less frequent but are among the most toxic of the DNA damage types due to their potential to disrupt chromosomal integrity. Therefore, DSB must be repaired to maintain chromosomal integrity.

Organisms have evolved several strategies to repair DSBs, which can be classified based on the nature of the break: two–ended and one–ended DSBs [24].

Two–ended DSBs are typically induced by site–specific nucleases, ionizing radiation or other damaging agents. The primary repair pathways include the following:

(1) Homologous recombination (HR): HR utilizes sister chromatids as templates to repair DSBs and is considered the most accurate method of repair. HR requires a complex formed by MRE11–RAD50–NBS1 (MRN) and the CtBP–interacting protein (CtIP). The complex initiates short–range DNA end resection, forming 3′–terminated ssDNA, and then recruits exonuclease 1 (EXO1) or the DNA replication ATP–dependent helicase/nuclease 2 (DNA2) and Bloom syndrome protein (BLM) or Wemer syndrome protein (WRN) and other necessary factors to complete long–range end resection. This process generates longer single–stranded DNA (ssDNA) to facilitate recombination, which is conducive to accurate repair. Next, HR involves gene conversion (GC) and may result in synthesis–dependent strand annealing (SDSA) or a double Holliday junction (dHJ). During the SDSA process, one end of the DSB invades the homologous gene template and initiates replication from the donor. Subsequently, the DSB unwinds from its template to release the newly synthesized DNA strand. Then, the DSB anneals with the complementary DNA at the break sites and serves as a template for synthesizing the second strand, thereby completing the DNA replication process. The initiation of dHJ is similar to that of SDSA, but the D–loop “captures” the second broken DNA strand via annealing, thus forming the Holliday junction. The Holliday junction determines the outcomes of crossovers and non–crossovers [25].
(2) Non-homologous end joining (NHEJ): NHEJ directly joins the two ends of the break, which can easily lead to base deletions or introduce some DNA fragment insertions. As a result, its error–proness rate greatly increases. Because NHEJ repair is fast and efficient, it occurs at various stages of the cell cycle and becomes the primary method of cell repair [26].
(3) Microhomology–mediated end joining (MMEJ): Firstly, MMEJ requires limited DNA end resection to expose short ssDNAs. Secondly, the microhomology DNA strands are annealed, or single–stranded DNA overhangs (Flaps) need to be removed. Lastly, gaps are populated, and DNAs are ligated [27].
(4) Single–strand annealing (SSA): Compared with MMEJ, SSA requires end resection to generate a longer 3′ ssDNA tail, which is then annealed between homologous regions. The repair is completed by removing the 3′ flaps, filling the gaps, and ligating the DNA [24].

One–ended DSBs typically arise from stalled replication forks, replications across ssDNA gaps, or severe telomere shortening. Key repair mechanisms include the following:

(1) Break–induced replication (BIR): BIR needs to undergo end resection to form a 3′ ssDNA that invades the homologous donor and then initiates bubble migration DNA synthesis, which can travel to the end of the chromosome [24].
(2) Mitotic DNA synthesis (MiDAS): A BIR–like process, MiDAS is observed at common fragile sites, where the DNA frequently remains unreplicated after the S phase [28].
(3) Reversed fork: Reversed forks, formed at stalled replication forks [29], involve the reconstitution of replication fork DNA. The newly synthesized strands may become uncoupled under replication stress, leading to the formation of a “chicken foot” structure as parent strands anneal [30]. When a reversed fork is inappropriately formed, it creates a one–ended DSB that is vulnerable to nuclease degradation. Intriguingly, it has been reported that the nucleases that resect and degrade stalled replication forks include MRE11, EXO1, and DNA2, as well as the nucleases that promote the resection of DSB ends. This degradation, also known as DNA end resection, can be inhibited by the DSB repair–independent functions of certain common DNA repair proteins. These proteins block the deleterious reversed fork from further degradation [31].

In the next section, we examine how the biochemical properties of HELQ contribute to its function in DSB repair.

### 4.2. HELQ Promotes End Resection in Resection–Dependent DSB Repair Pathways

In the early stages of DSB repair, DNA end resection plays a crucial role. According to whether DSB end resection is dependent or not, DSB repair can be divided into HR, SSA, MMEJ, and BIR, which rely on end resection and NHEJ, which does not involve end resection [32,33,34,35]. Another crucial function of DNA end resection is to regulate the selection of DSB repair pathways, inhibit DNA end resection, and contribute to NHEJ repair [36]. The dysregulation of end resection can result in genome instability, jeopardizing cellular functions and survival.

End resection typically proceeds in two phases: short–range resection and long–range resection. Initial resection at the DSB site is facilitated by CtIP in collaboration with the MRN complex, generating a short 3′ overhang ssDNA [37,38,39,40]. Although this short 3′ ssDNA is insufficient for strand invasion and HR, it is crucial for recruiting long–range end–resection factors like DNA2, EXO1, BLM, and WRN [33,41]. The MRN complex and CtIP are fundamental to initiating this process.

Regarding the second step of resection, the classical model previously considered that there are at least two pathways. One mechanism involves EXO1, which can independently cleave dsDNA and expose ssDNA with dsDNA–specific 5′–3′ exonuclease activity, and the other is accomplished by DNA2 in conjunction with a helicase. DNA2 possesses an ssDNA–specific 5′–3′ exonuclease activity which can cleave the ssDNA unwound by the helicase BLM or WRN to jointly complete the end resection and expose the ssDNA. In the classical model, EXO1, DNA2, BLM, and WRN are considered to be the core proteins responsible for driving long–range end resection [34,37,39,42,43,44]. While the EXO1 and DNA2/BLM/WRN complexes have proven critical for long–range resection, they require the MRN complex to initiate this process [45].

Recently, a research team led by Professor Kong Daochun at Peking University has made a significant discovery. Their latest study, published online in *Cell* [46], indicates that MRN facilitates the recruitment of RNAPIII (RNA polymerase III) to the DSB site. Subsequently, after MRN/CtIP generates a short ssDNA, RNAPIII utilizes the exposed ssDNA as a template to synthesize RNA strands, leading to the formation of an RNA–DNA hybrid structure. This hybrid structure protects the ssDNA produced by end resection. At the same time, RNAPIII also promotes the untwisting of dsDNA during RNA synthesis, forming a 3′–flap structure that is favored by DNA2. This structure can facilitate the end resection by DNA2. In summary, MRN/CtIP, EXO1, DNA2, BLM, and WRN form a small complex that effectively regulates DNA end resection. Many other helicases and nuclease factors have been reported to regulate end resection through direct or indirect pathways. 

**Figure 3:**
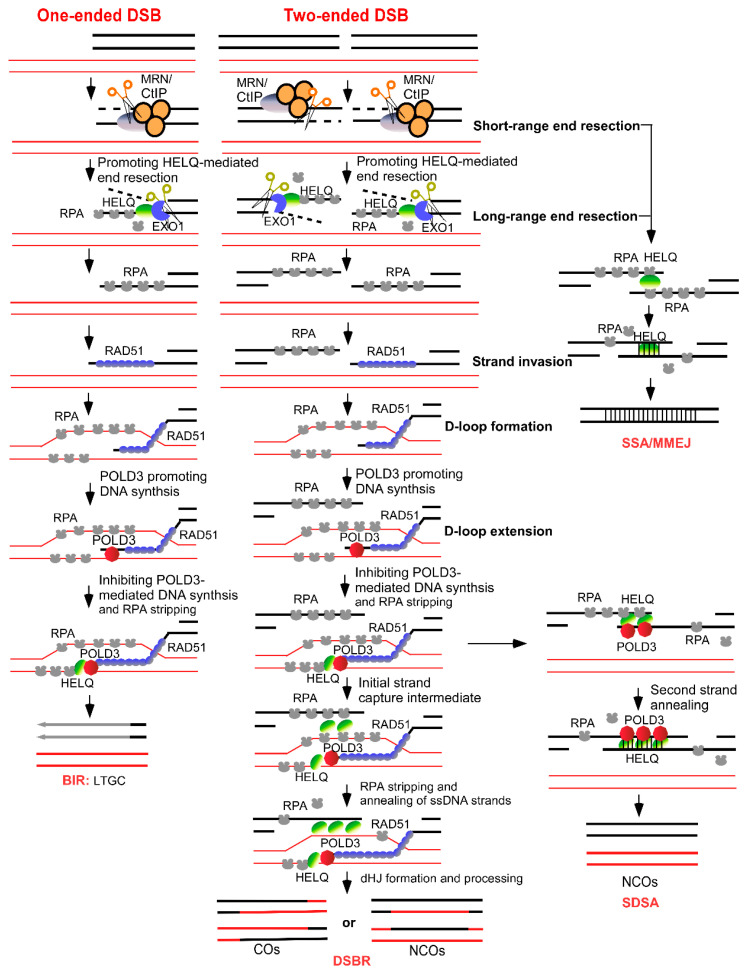
HELQ function in different DSB repairs including HR (SDSA and DSBR are the sub-pathway of HR), BIR, SSA, and MMEJ. In the early stage, HELQ promotes end resection after DSB occurs. In the late stage, after D–loop formation, HELQ might inhibit POLD3–mediated DNA synthesis to avoid a large number of genetic recombinations and gene conversions in BIR and HR repair, and, moreover, HELQ strips RPA from DNAs conducive to ssDNA annealing in DSB repair. Red line: template sister chromatid; black line: recipient chromatid; dash line: resected DNA.

HELQ, a helicase implicated in DNA repair, also plays a significant role in end resection (Figure 3). Initially, Anand et al. suggested that HELQ could promote resection–dependent DSB repair and slightly inhibit NHEJ [8]. However, their conclusions, based on RPA2 phosphorylation as an indirect marker of resection, might have underestimated HELQ’s role. The prolonged drug treatment used in their study depleted RPA2, leaving its phosphorylation levels unchanged in HELQ knockout cells compared with wild–type cells. This could have obscured HELQ’s contribution to end resection.

The latest research results demonstrate that HELQ can enhance end resection in the typical DSB repair pathway [9]. HELQ is primarily recruited to DSB damage sites through its ssDNA–binding activity, which promotes nuclease EXO1–mediated end resection, which is conducive to HR repair. Although its helicase activity can also promote end resection, it is far less pronounced than its ssDNA–binding ability. This difference may stem from its requirement to bind to ssDNA, which is crucial for promoting resection. HELQ mutants with impaired helicase activity still maintain the ability to bind to ssDNA or dsDNA. Additionally, it has been reported that RPA inhibits the activity of EXO1. HELQ mutants with impaired helicase activity are unable to displace RPA from the ssDNA strand, leading to only a partial impairment of the resection. Further experimental studies are required to prove whether the translocation activity and DNA–strand–annealing activity of HELQ can assist in end resection.

The identification of HELQ emerged from efforts by Wood et al. to discover human proteins involved in the repair of DNA interstrand cross–links (ICLs) [4]. It was later confirmed that HELQ does have a role in Drosophila, Caenorhabditis elegans, or mammalian tumor cells, demonstrating its significance in participating in ICL [3,4,47,48,49,50]. ICLs are deleterious DNA lesions caused by endogenous sources (malondialdehyde) or exogenous sources (mitomycin C (MMC), cisplatin, nitrogen mustard, and psoralens), which induce mutations and chromosomal rearrangements by inhibiting DNA replication and transcription. Since ICL repair necessitates the coordinated action of checkpoints and Fanconi anemia (FA) pathways during the synthesis phase (S), it facilitates ICL cleavage, translesion DNA synthesis (TLS), and HR [51,52]. Given that HR repair is integral to ICL repair, it is plausible that HELQ’s sensitivity to DNA crosslinkers is linked to its role in these repair processes.

Evidence suggests that HELQ may facilitate ICL repair through mechanisms parallel to those involving FANCD2, which is a central component of the FA pathway [48,49,50]. However, further investigation into the genetic interactions between HELQ and FANCD2, using HR repair reporter cell lines and ISCE1 endonuclease–induced DSB models, has revealed that both proteins are indeed part of the same repair pathway [12]. Despite this, the precise regulatory mechanisms through which HELQ contributes to ICL repair remain unclear and warrant further elucidation. Thus, ongoing research is essential to fully delineate HELQ’s role and its interplay with other key repair factors in the context of ICL damage.

### 4.3. HELQ Inhibits Nascent DNA Degradation (End Resection) in Reversed Forks

According to the literature, certain protein factors involved in the HR process, such as BRCA1, BRCA2, CtIP, and RAD51, have been shown to protect the one–ended DSBs formed by replication fork reversal. These protein factors also prevent the degradation of replication forks by MRE11, EXO1, DNA2, and other resection factors [29,31,35,53,54,55,56].

Notably, HELQ, a helicase known for its involvement in DSB end resection, has also been implicated in the stabilization of reversed replication forks. Firstly, HELQ acts as a helicase and has been found to prefer unwound forked–DNA substrates in vitro [4,8,18]. Moreover, HELQ can inhibit a stalling replication fork collapse in mammalian cells. Relying on ssDNA–binding activities instead of helicase activities to recruit to the reversed fork [9], HELQ inhibits the cleavage of nascent DNA on the reversed fork by the nucleases MRE11, DNA2, and MUS81. HELQ achieves this by stabilizing the RAD51 nucleofilaments bound to the nascent DNA on the stalled replication fork. HELQ and CtIP act in different pathways to stabilize the binding of RAD51 to nascent DNAs on the arrested replication fork. Moreover, HELQ and CtIP synergistically protect the arrested replication fork and maintain its stability, rather than with BRCA1 or BRCA2.

**Figure 4:**
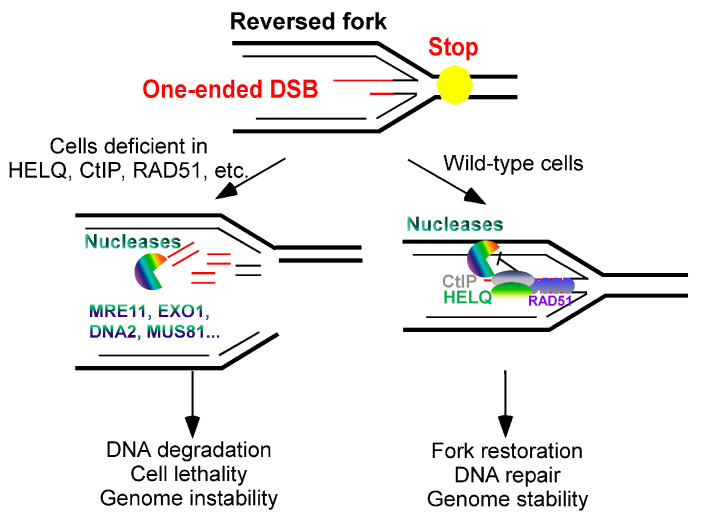
HELQ function in reversed forks. HELQ synthesizes with CtIP to stabilize RAD51 nucleoprotein filaments to protect stalling replication forks from nuclease degradation DNA and maintain genome stability. Red line: nascent DNA; black line: non-nascent DNA.

The combined deletion of HELQ and CtIP results in heightened sensitivity to replication stress and exhibits synergistic lethal effects on tumor cells [9]. This observation indicates that HELQ and CtIP address endogenous replication pressure through complementary mechanisms (Figure 4). Despite these insights, the full extent of HELQ’s regulatory role in one–ended DSBs remains only partially understood. Further experimental studies are necessary to elucidate whether HELQ’s translocation and DNA–strand–annealing activities also contribute to its protective function at reversed forks.

### 4.4. The Function of HELQ in Recombination

In addition to facilitating DNA end resection, HELQ is also considered useful for the downstream events of resection (Figure 3). After the resection is completed in the HR repair pathway, RPA needs to bind to ssDNA to protect the DNA. Subsequently, RAD51 mediates invasion, which necessitates the displacement of RPA. This is followed by strand annealing to facilitate DNA recombination and ultimately complete the repair process [25]. Research led by Simon J. Boulton elucidates that HELQ participates in DSB repair by modulating the functions of RPA and RAD51. Through biochemical and single–molecule imaging analyses, it was demonstrated that HELQ interacts with RAD51 during DNA unwinding, significantly stimulating HELQ’s translocation activity. Conversely, RPA inhibits HELQ’s ability to unwind DNA while strongly promoting its strand–annealing activity. Mechanistically, HELQ facilitates DNA unwinding with RAD51’s assistance, captures ssDNAs bound to RPA, and promotes the annealing of complementary DNA sequences [8].

Based on these findings, it has been further speculated that HELQ regulates DSB repair in mammalian cells. In various DSB reporter cell lines, HELQ deletion impairs the HR, SSA, and MMEJ repair pathways, leading to a preference for long–fragment gene switching during HR [8]. This suggests that HELQ plays a role in multiple steps of HR or SDSA by modulating its intrinsic binding, translocation, unwinding, and annealing activities in conjunction with RPA and RAD51. However, SSA repair, which relies on long ssDNA fragments exposed by end resection and does not involve RAD51, may primarily engage HELQ’s DNA–binding and –annealing activities (Figure 3). For MMEJ repair, which requires only limited resection, HELQ’s role involves removing RPA from ssDNAs, a process crucial for facilitating the annealing of microhomology sequences. Thus, HELQ’s function in MMEJ likely necessitates both its DNA–binding and –annealing capabilities. The activity of HELQ across different DSB repair pathways is intricately guided by complementary base pairs. Future research is expected to provide more definitive data on HELQ’s comprehensive roles in these repair processes.

The key step in HR is the formation of a displacement loop (D–loop) by a RecA–family recombinase. This enzyme catalyzes the invasion of an unbroken duplex by the broken DNA strand, initiating a new DNA synthesis from the 3′OH end that is available. DNA synthesis in this context is eventually halted, and the chromosome is reconstituted via annealing and ligation of nascent DNAs—a non–crossover outcome to recombination—or by the resolution of branched DNA intermediates to produce a crossover outcome [25].

In studies with the archaeal model organism *Archaeoglobus fulgidus*, Lever et al. [10] demonstrated that purified Hel308 exhibits hyperactive DNA–helicase and –annealing enzyme activities in vitro through a single amino acid substitution in the motif IVa of the RecA2 domain. Motif IVa regulates the unwinding and annealing processes of Hel308. The IVa mutation in HEL308 results in a 160,000–fold increase in genetic recombination, predominantly through gene conversion (non-crossover) events. However, cross–recombination is unaffected by mutations in motif IVa, nor cell viability or DNA damage susceptibility. In contrast, Hel308 deficiency results in stunted growth, increased sensitivity to DNA crosslinkers, and only a slight increase in the recombination rate [10]. These findings suggest that the archaeal organism *Archaeoglobus fungus* Hel308 repairs DNA damage by avoiding extensive genetic recombination and gene conversion. Therefore, it is reasonable to speculate that the ssDNA–binding activity of HELQ should also inhibit genetic recombination and gene conversion, thereby facilitating DSB repair. Further verification is required regarding the translocation ability and suitability of HELQ for mammalian cells.

### 4.5. HELQ Function in BIR Repair

BIR is a crucial repair pathway for one–ended DSBs and involves end resection, akin to HR. BIR is distinct in that it is initiated by RAD52 and RAD51, which facilitate the invasion of a broken DNA end into a homologous template. A critical player in BIR is POLD3, a subunit of DNA polymerase delta (Pol δ), which is essential for synthesizing new DNAs within a migrating bubble. This process ensures the conservative inheritance of genetic material and is distinct from the DNA synthesis occurring during the S phase of the cell cycle [24]. Pol δ, especially its catalytic subunit POLD1, plays a pivotal role in DNA synthesis within eukaryotic DSB repair pathways such as BIR. A proper regulation of Pol δ activity is crucial to prevent genomic instability [24]. Liu He et al. discovered that HelQ/HELQ restricts DNA synthesis by physically interacting with POLD3, rather than with the POLD1 subunit of Pol δ. HelQ interacts with POLD3 using approximately 70 amino acids (amino acids 1–76) in its N–terminal disordered region, which is distinct from the RPA–binding region (Asp–141 and Phe–142 residues of HELQ PWI), thereby possibly strongly stimulating HelQ–mediated DNA single–stranded annealing and promoting homology–dependent repair pathways such as BIR, microhomology–mediated BIR (MM–BIR), and SDSA to uphold genomic stability [22]. Furthermore, BIR, or MM–BIR triggered by DNA replication, leads to genetic rearrangements, tandem duplications, and mutagenesis, which are characteristic of cells under chronic DNA damage and replication stress [24]. This could explain why the loss of helq-1 leads to the spontaneous accumulation of tandem duplications in the genomes of helq-1 mutants in *Caenorhabditis elegans* [57].

A BIR–like process recently identified in mammalian cells is mitotic DNA synthesis (MiDAS), which is initiated at common fragile sites (CFSs) and regions of the genome prone to remain unreplicated after the S phase [24]. The timely resolution of late replication intermediates (LRIs) is essential for faithful genome duplication, especially under replication stress. In human cancer cells, MiDAS is considered a final mechanism to resolve LRIs and prevent lethal chromosome mis–segregation. RAD52–driven MiDAS accomplishes this by creating gaps or breaks on metaphase chromosomes, particularly at CFSs [28,58]. Primary human cells exhibit RAD52–independent MiDAS, yet FANCD2 interdependence with HELQ–driven MiDAS is crucial for safeguarding CFS stability early in replication. Conversely, RAD52–driven MiDAS in cancer cells may be adapted to prevent chromosome mis-segregation, potentially impacting CFS expression, unlike the synergistic effect of HELQ and FANCD2 in promoting ICL repair [49,59]. HELQ and FANCD2 promote MiDAS in a genetically epistatic manner, however, with FANCD2 acting in parallel with POLD3 [60]. HELQ could inhibit POLD3–mediated DNA synthesis. Therefore, based on our literature review, the relationship between HELQ and POLD3 in regulating MiDAS in primary human cells is undetermined. The reviewed studies only reported the function of HELQ in MiDAS but did not explore how the biochemical activity of HELQ affects MiDAS.

In summary, HELQ activity supports all DSB repair pathways involving complementary base pairs, encompassing multiple steps crucial for maintaining genome stability.

## 5. The Role of HELQ in Human Diseases

HELQ plays a crucial role in inhibiting the onset and progression of ovarian cancer. Recent studies have illuminated HELQ’s pivotal role not only in ovarian cancer but also across a spectrum of other cancer types. Genetic alterations in HELQ frequently occur in various cancers, including oral squamous cell carcinoma, hepatocellular carcinoma, pancreatic ductal adenocarcinoma, gastric cancer, upper gastrointestinal cancer, head and neck squamous cell carcinoma, and ovarian cancer [1].

Adelman et al. utilized mice as a model organism to introduce a β–Geo–established Helq–deficient mouse using gene trap technology. They demonstrated heightened sensitivity to pituitary tumors, gastric cancer, and ovarian cancer. Additionally, this deficiency was linked to early menopause in lactating animals [48]. Helq gene deletion is identified as the most prevalent genetic variant, occurring in 54% of ovarian cancer cases [1]. The results of a structure–function analysis and an association with RAD51 paralogs suggest that HELQ is a sporadic or hereditary candidate for the ovarian oncogene [61,62]. HELQ overexpression has been linked to an increased resistance to cisplatin, enhanced DNA repair activity, and elevated expression of DNA repair proteins in the nucleotide excision repair (NER) pathway. Conversely, HELQ knockdown results in the opposite effects, positioning HELQ as a potential novel biomarker for chemoresistance in epithelial ovarian cancer (EOC) and a prognostic predictor [63]. HELQ’s role in HR repair has been implicated in the development and progression of endometrial stromal sarcoma (ESS) and other endometrial malignancies [64]. Zhu et al. determined that HELQ and XAB2 expression levels could potentially predict primary chemoresponsiveness and prognosis in high–grade serous carcinoma (HGSC) [65]. Epidemiological studies have shown significant associations of HEL308 with head and neck squamous cell carcinoma (HNCSC), particularly among smokers [66]. Liu et al. demonstrated that HELQ confers an anti-invasion phenotype to osteosarcoma (OS) cells by activating the CHK1 signaling pathway, suggesting that HELQ is a promising therapeutic target for OS cells and other malignancies [67]. Additionally, HELQ and EGR3 expression levels were associated with the mutation status of the heavy chain variable region (IgHV) in patients with chronic lymphocytic leukemia (CLL), indicating that HELQ/EGR3 is a potential prognostic marker linked to targeted cell signaling pathways [68].

Overall, HELQ’s role appears pivotal in inhibiting the occurrence and progression of ovarian cancer. Therefore, conducting detailed research on HELQ’s mechanism of action in DSB repair may hold promise for advancing ovarian cancer treatment strategies. The involvement of HELQ in DSB repair is particularly significant, as DSBs are one of the most deleterious forms of DNA damage, leading to genomic instability and tumorigenesis if not properly repaired. HELQ’s function in facilitating various DSB repair pathways, including HR, MMEJ, SSA, and BIR, and protecting reversed forks, highlights its essential role in maintaining genomic integrity. Understanding these mechanisms provides a foundation for exploring HELQ as a therapeutic target not only in ovarian cancer but also in other malignancies where DSB repair is compromised. By targeting HELQ and modulating its activity, new therapeutic approaches could be developed to enhance the efficacy of existing treatments and potentially prevent the onset of cancer in high–risk individuals.

## 6. Conclusions and Outlook

In this review, an in-depth study of HELQ from various perspectives was conducted to elucidate the structural characteristics of HELQ. Through a multi–species sequence alignment, an analysis of the atomic resolution structure of archaeal HELQ, a prediction of the human HELQ structure using AlphaFold, and a verification of biochemical activities in vitro and in vivo, the ssDNA–binding site and –helicase activity site have been identified. However, the translocating sites and DNA–annealing–activity sites remain undetermined. Marini et al.’s speculation that it may have a polymeric form remains to be resolved [4]. HELQ is able to participate in a wide variety of DSB repair pathways and involves multiple steps of a specific DSB repair process. Therefore, it is also important to clarify the biological significance of HELQ’s various biochemical activities and its polymeric or monomeric structure. Currently, numerous studies have shown that HELQ primarily plays a crucial role in inhibiting the occurrence and progression of ovarian cancer, and it also exhibits partial effects in some other types of cancer. Addressing these questions could lead to targeted therapies for tumors associated with HELQ and its mutations, such as ovarian cancer. For example, based on HELQ’s reported ssDNA–binding ability, it is crucial for end resection at DSB sites and stalled replication forks [9]. In future research, we will actively engage in the development of small molecule inhibitors targeting the HELQ K587 site to inhibit the ssDNA–binding activity of HELQ. This development will aid in clinically treating breast cancer patients with CtIP mutations that lead to the deletion of replication–fork protection. Conversely, it is necessary to identify the loci of high–frequency HELQ mutations in the clinical tissue samples of various cancer types (e.g., breast cancer patients) in the TCGA database or even in patients detecting end resection in the DSB sites and protect against stalled replication forks. This will also facilitate the clinical treatment of patients with HELQ mutations by developing specific CtIP inhibitors. A high expression of HELQ in patients with high–grade serous ovarian cancer and breast cancer is associated with a poor prognosis [65]. Therefore, investigating the functional abnormalities of HELQ that contribute to this poor prognosis will be beneficial for the development of anti-tumor–targeted drugs. Finally, exploring the corresponding DSB repair function of HELQ based on its biochemical activity and molecular structure characteristics is of great significance for predicting certain tumors or developing anti–tumor–targeted drugs.

## References

[1] Han X., Zhao L., Li X. (2016). HELQ in cancer and reproduction. Neoplasma.

[2] Boyd J.B., Sakaguchi K., Harris P.V. (1990). mus308 mutants of Drosophila exhibit hypersensitivity to DNA cross-linking agents and are defective in a deoxyribonuclease. Genetics.

[3] Harris P.V., Mazina O.M., Leonhardt E.A., Case R.B., Boyd J.B., Burtis K.C. (1996). Molecular cloning of Drosophila mus308, a gene involved in DNA cross-link repair with homology to prokaryotic DNA polymerase I genes. Mol. Cell Biol..

[4] Marini F., Wood R.D. (2002). A human DNA helicase homologous to the DNA cross-link sensitivity protein Mus308. J. Biol. Chem..

[5] Fujikane R., Komori K., Shinagawa H., Ishino Y. (2005). Identification of a novel helicase activity unwinding branched DNAs from the hyperthermophilic archaeon, *Pyrococcus furiosus*. J. Biol. Chem..

[6] Guy C.P., Bolt E.L. (2005). Archaeal Hel308 helicase targets replication forks in vivo and in vitro and unwinds lagging strands. Nucleic Acids Res..

[7] Richards J.D., Johnson K.A., Liu H., McRobbie A.M., McMahon S., Oke M., Carter L., Naismith J.H., White M.F. (2008). Structure of the DNA repair helicase hel308 reveals DNA binding and autoinhibitory domains. J. Biol. Chem..

[8] Anand R., Buechelmaier E., Belan O., Newton M., Vancevska A., Kaczmarczyk A., Takaki T., Rueda D.S., Powell S.N., Boulton S.J. (2022). HELQ is a dual-function DSB repair enzyme modulated by RPA and RAD51. Nature.

[9] Zhao Y.Q., Hou K.P., Li Y.H., Hao S.L., Liu Y., Na Y.A., Li C., Cui J., Xu X.Z., Wu X.H. (2023). Human HELQ regulates DNA end resection at DNA double-strand breaks and stalled replication forks. Nucleic Acids Res..

[10] Lever R.J., Simmons E., Gamble-Milner R., Buckley R.J., Harrison C., Parkes A.J., Mitchell L., Gausden J.A., Skulj S., Bertosa B. (2023). Archaeal Hel308 suppresses recombination through a catalytic switch that controls DNA annealing. Nucleic Acids Res..

[11] Tang N., Wen W., Liu Z., Xiong X., Wu Y. (2023). HELQ as a DNA helicase: Its novel role in normal cell function and tumorigenesis (Review). Oncol. Rep..

[12] Moldovan G.L., Madhavan M.V., Mirchandani K.D., McCaffrey R.M., Vinciguerra P., D’Andrea A.D. (2010). DNA polymerase POLN participates in cross-link repair and homologous recombination. Mol. Cell Biol..

[13] Buttner K., Nehring S., Hopfner K.P. (2007). Structural basis for DNA duplex separation by a superfamily-2 helicase. Nat. Struct. Mol. Biol..

[14] Oyama T., Oka H., Mayanagi K., Shirai T., Matoba K., Fujikane R., Ishino Y., Morikawa K. (2009). Atomic structures and functional implications of the archaeal RecQ-like helicase Hjm. BMC Struct. Biol..

[15] Northall S.J., Buckley R., Jones N., Penedo J.C., Soultanas P., Bolt E.L. (2017). DNA binding and unwinding by Hel308 helicase requires dual functions of a winged helix domain. DNA Repair.

[16] Flechsig H., Popp D., Mikhailov A.S. (2011). In silico investigation of conformational motions in superfamily 2 helicase proteins. PLoS ONE.

[17] Jenkins T., Northall S.J., Ptchelkine D., Lever R., Cubbon A., Betts H., Taresco V., Cooper C.D.O., McHugh P.J., Soultanas P. (2021). The HelQ human DNA repair helicase utilizes a PWI-like domain for DNA loading through interaction with RPA, triggering DNA unwinding by the HelQ helicase core. NAR Cancer.

[18] Tafel A.A., Wu L., McHugh P.J. (2011). Human HEL308 localizes to damaged replication forks and unwinds lagging strand structures. J. Biol. Chem..

[19] Craig J.M., Laszlo A.H., Nova I.C., Brinkerhoff H., Noakes M.T., Baker K.S., Bowman J.L., Higinbotham H.R., Mount J.W., Gundlach J.H. (2019). Determining the effects of DNA sequence on Hel308 helicase translocation along single-stranded DNA using nanopore tweezers. Nucleic Acids Res..

[20] Brinkerhoff H., Kang A.S.W., Liu J., Aksimentiev A., Dekker C. (2021). Multiple rereads of single proteins at single-amino acid resolution using nanopores. Science.

[21] Li Z., Lu S., Hou G., Ma X., Sheng D., Ni J., Shen Y. (2008). Hjm/Hel308A DNA helicase from Sulfolobus tokodaii promotes replication fork regression and interacts with Hjc endonuclease in vitro. J. Bacteriol..

[22] He L., Lever R., Cubbon A., Tehseen M., Jenkins T., Nottingham A.O., Horton A., Betts H., Fisher M., Hamdan S.M. (2023). Interaction of human HelQ with DNA polymerase delta halts DNA synthesis and stimulates DNA single-strand annealing. Nucleic Acids Res..

[23] Chatterjee N., Walker G.C. (2017). Mechanisms of DNA damage, repair, and mutagenesis. Environ. Mol. Mutagen..

[24] Kockler Z.W., Osia B., Lee R., Musmaker K., Malkova A. (2021). Repair of DNA Breaks by Break-Induced Replication. Annu. Rev. Biochem..

[25] Li X., Heyer W.D. (2008). Homologous recombination in DNA repair and DNA damage tolerance. Cell Res..

[26] Chang H.H.Y., Pannunzio N.R., Adachi N., Lieber M.R. (2017). Non-homologous DNA end joining and alternative pathways to double-strand break repair. Nat. Rev. Mol. Cell Biol..

[27] Wang H., Xu X. (2017). Microhomology-mediated end joining: New players join the team. Cell Biosci..

[28] Minocherhomji S., Ying S., Bjerregaard V.A., Bursomanno S., Aleliunaite A., Wu W., Mankouri H.W., Shen H., Liu Y., Hickson I.D. (2015). Replication stress activates DNA repair synthesis in mitosis. Nature.

[29] Berti M., Cortez D., Lopes M. (2020). The plasticity of DNA replication forks in response to clinically relevant genotoxic stress. Nat. Rev. Mol. Cell Biol..

[30] Quinet A., Lemacon D., Vindigni A. (2017). Replication Fork Reversal: Players and Guardians. Mol. Cell.

[31] Cejka P., Symington L.S. (2021). DNA End Resection: Mechanism and Control. Annu. Rev. Genet..

[32] Cejka P. (2015). DNA End Resection: Nucleases Team Up with the Right Partners to Initiate Homologous Recombination. J. Biol. Chem..

[33] Symington L.S. (2016). Mechanism and regulation of DNA end resection in eukaryotes. Crit. Rev. Biochem. Mol. Biol..

[34] Ranjha L., Howard S.M., Cejka P. (2018). Main steps in DNA double-strand break repair: An introduction to homologous recombination and related processes. Chromosoma.

[35] Zhao F., Kim W., Kloeber J.A., Lou Z. (2020). DNA end resection and its role in DNA replication and DSB repair choice in mammalian cells. Exp. Mol. Med..

[36] Symington L.S., Gautier J. (2011). Double-strand break end resection and repair pathway choice. Annu. Rev. Genet..

[37] Longhese M.P., Bonetti D., Manfrini N., Clerici M. (2010). Mechanisms and regulation of DNA end resection. EMBO J..

[38] Mimitou E.P., Symington L.S. (2011). DNA end resection--unraveling the tail. DNA Repair.

[39] Nimonkar A.V., Genschel J., Kinoshita E., Polaczek P., Campbell J.L., Wyman C., Modrich P., Kowalczykowski S.C. (2011). BLM-DNA2-RPA-MRN and EXO1-BLM-RPA-MRN constitute two DNA end resection machineries for human DNA break repair. Genes. Dev..

[40] Yun M.H., Hiom K. (2009). CtIP-BRCA1 modulates the choice of DNA double-strand-break repair pathway throughout the cell cycle. Nature.

[41] Huertas P., Jackson S.P. (2009). Human CtIP mediates cell cycle control of DNA end resection and double strand break repair. J. Biol. Chem..

[42] Ceppi I., Howard S.M., Kasaciunaite K., Pinto C., Anand R., Seidel R., Cejka P. (2020). CtIP promotes the motor activity of DNA2 to accelerate long-range DNA end resection. Proc. Natl. Acad. Sci. USA.

[43] Gravel S., Chapman J.R., Magill C., Jackson S.P. (2008). DNA helicases Sgs1 and BLM promote DNA double-strand break resection. Genes. Dev..

[44] Symington L.S. (2014). End resection at double-strand breaks: Mechanism and regulation. Cold Spring Harb. Perspect. Biol..

[45] Sturzenegger A., Burdova K., Kanagaraj R., Levikova M., Pinto C., Cejka P., Janscak P. (2014). DNA2 cooperates with the WRN and BLM RecQ helicases to mediate long-range DNA end resection in human cells. J. Biol. Chem..

[46] Liu S., Hua Y., Wang J., Li L., Yuan J., Zhang B., Wang Z., Ji J., Kong D. (2021). RNA polymerase III is required for the repair of DNA double-strand breaks by homologous recombination. Cell.

[47] Muzzini D.M., Plevani P., Boulton S.J., Cassata G., Marini F. (2008). Caenorhabditis elegans POLQ-1 and HEL-308 function in two distinct DNA interstrand cross-link repair pathways. DNA Repair.

[48] Adelman C.A., Lolo R.L., Birkbak N.J., Murina O., Matsuzaki K., Horejsi Z., Parmar K., Borel V., Skehel J.M., Stamp G. (2013). HELQ promotes RAD51 paralogue-dependent repair to avert germ cell loss and tumorigenesis. Nature.

[49] Takata K., Reh S., Tomida J., Person M.D., Wood R.D. (2013). Human DNA helicase HELQ participates in DNA interstrand crosslink tolerance with ATR and RAD51 paralogs. Nat. Commun..

[50] Luebben S.W., Kawabata T., Akre M.K., Lee W.L., Johnson C.S., O’Sullivan M.G., Shima N. (2013). Helq acts in parallel to Fancc to suppress replication-associated genome instability. Nucleic Acids Res..

[51] Jones M.J., Huang T.T. (2012). The Fanconi anemia pathway in replication stress and DNA crosslink repair. Cell Mol. Life Sci..

[52] Hashimoto S., Anai H., Hanada K. (2016). Mechanisms of interstrand DNA crosslink repair and human disorders. Genes. Environ..

[53] Liao H., Ji F., Helleday T., Ying S. (2018). Mechanisms for stalled replication fork stabilization: New targets for synthetic lethality strategies in cancer treatments. EMBO Rep..

[54] Rickman K., Smogorzewska A. (2019). Advances in understanding DNA processing and protection at stalled replication forks. J. Cell Biol..

[55] Kondratick C.M., Washington M.T., Spies M. (2021). Making Choices: DNA Replication Fork Recovery Mechanisms. Semin. Cell Dev. Biol..

[56] Thakar T., Moldovan G.L. (2021). The emerging determinants of replication fork stability. Nucleic Acids Res..

[57] Kamp J.A., Lemmens B., Romeijn R.J., Changoer S.C., van Schendel R., Tijsterman M. (2021). Helicase Q promotes homology-driven DNA double-strand break repair and prevents tandem duplications. Nat. Commun..

[58] Bhowmick R., Minocherhomji S., Hickson I.D. (2016). RAD52 Facilitates Mitotic DNA Synthesis Following Replication Stress. Mol. Cell.

[59] Wang W., Zhao S., Zhuang L., Li W., Qin Y., Chen Z.J. (2015). The screening of HELQ gene in Chinese patients with premature ovarian failure. Reprod. Biomed. Online.

[60] Traband E.L., Hammerlund S.R., Shameem M., Narayan A., Ramana S., Tella A., Sobeck A., Shima N. (2023). Mitotic DNA Synthesis in Untransformed Human Cells Preserves Common Fragile Site Stability via a FANCD2-Driven Mechanism That Requires HELQ. J. Mol. Biol..

[61] Li Y.P., Yang J.J., Xu H., Guo E.Y., Yu Y. (2016). Structure-function analysis of DNA helicase HELQ: A new diagnostic marker in ovarian cancer. Oncol. Lett..

[62] Pelttari L.M., Kinnunen L., Kiiski J.I., Khan S., Blomqvist C., Aittomaki K., Nevanlinna H. (2016). Screening of HELQ in breast and ovarian cancer families. Fam. Cancer.

[63] Long J., Zhu J.Y., Liu Y.B., Fu K., Tian Y., Li P.Y., Yang W.Q., Yang S.Y., Yin J.Y., Yin G. (2018). Helicase POLQ-like (HELQ) as a novel indicator of platinum-based chemoresistance for epithelial ovarian cancer. Gynecol. Oncol..

[64] Liu Y., Zhang Y., Tian Y. (2020). Expressions of HELQ and RAD51C in endometrial stromal sarcoma and their clinical significance. Nan Fang. Yi Ke Da Xue Xue Bao.

[65] Zhu F., Yang S., Lei M., He Q., Wu L., Zhang Y. (2022). DNA Repair Protein HELQ and XAB2 as Chemoresponse and Prognosis Biomarkers in Ascites Tumor Cells of High-Grade Serous Ovarian Cancer. J. Oncol..

[66] Liang C., Marsit C.J., Houseman E.A., Butler R., Nelson H.H., McClean M.D., Kelsey K.T. (2012). Gene-environment interactions of novel variants associated with head and neck cancer. Head. Neck.

[67] Liu D.N., Zhou Y.F., Peng A.F., Long X.H., Chen X.Y., Liu Z.L., Xia H. (2017). HELQ reverses the malignant phenotype of osteosarcoma cells via CHK1-RAD51 signaling pathway. Oncol. Rep..

[68] Guo C., Gao Y.Y., Ju Q.Q., Zhang C.X., Gong M., Li Z.L. (2021). HELQ and EGR3 expression correlate with IGHV mutation status and prognosis in chronic lymphocytic leukemia. J. Transl. Med..

